# The roles of binge gaming in social, academic and mental health outcomes and gender differences: A school-based survey in Hong Kong

**DOI:** 10.1371/journal.pone.0327365

**Published:** 2025-08-13

**Authors:** Nick Tse, Natalie Sze Nga Pang, Xin Wang, Yiran Li, Camilla Kin Ming Lo, Xue Yang

**Affiliations:** 1 Department of Applied Social Sciences, HKCT Institute of Higher Education, Hong Kong SAR, China; 2 The Jockey Club School of Public Health and Primary Care, Faculty of Medicine, Chinese University of Hong Kong, Hong Kong SAR, China; 3 Department of Applied Social Sciences, The Hong Kong Polytechnic University, Hong Kong SAR, China; Yale University, UNITED STATES OF AMERICA

## Abstract

Binge gaming, defined as playing video games for more than five consecutive hours, has become an emerging health and behavioral issue among children and adolescents. However, the potential factors and consequences have not been sufficiently investigated. This study aims to examine the prevalence of binge gaming, its social, academic, and mental health consequences, and potential gender differences among children and adolescents in Hong Kong. A school-based survey was conducted on 2,592 primary and secondary students in Hong Kong. The sample included 1,404 boys (mean age = 11.99 + /- 2.415 years) and 1,188 girls (mean age = 11.77 + /- 2.473 years), with an overall mean age of 11.89 years. Internet gaming disorder (IGD), depression, anxiety, stress, loneliness, social support, sleep quality, and educational self-efficacy were measured using well-validated self-report scales. Analysis of Covariance (ANCOVA) models, adjusting for age and daily gaming time, were employed to examine mental, social, and academic outcomes among binge gamers, non-binge gamers, and non-gamers. Subgroup analyses were conducted by gender. The overall prevalence of binge gaming in the study sample was 31.7%. Thirty percent of respondents reported at least one episode of binge gaming in the last month, with 38.3% in boys and 24.0% in girls. In boys, binge gamers showed greater IGD, depression, anxiety, and stress, poorer sleep quality, and lower educational self-efficacy than non-binge gamers. Similarly, female binge gamers exhibited higher IGD, depression, anxiety, stress, and loneliness, and lower educational self-efficacy, sleep quality, and social support. Non-gamers generally experienced less depression, anxiety, stress, and loneliness, and higher educational self-efficacy, than binge gamers in both genders. Binge gaming may act as a behavioral indicator and risk factor for various social, academic, and health problems and preventive effort is warranted.

## Introduction

Internet gaming has emerged as the most popular activity among young people, especially boys [1–3]. This surge in popularity can be attributed to the blend of entertainment and educational aspects inherent in these activities [4]. In a recent study in Hong Kong, most adolescents spent more than 4 hours per day on their smartphones (97%) and computers (91%) [5]. Nevertheless, a mounting body of evidence suggests that excessive gaming may manifest as pathological behaviours, potentially leading to internet gaming disorder (IGD) [6,7].

The latest edition of the Diagnostic Statistical Manual of Mental Disorders (DSM-5), released in 2013, included IGD in the supplementary section of “Conditions for further study” [8]. The International Classification of Diseases 11^th^ edition (ICD-11) officially recognized IGD as a mental disorder. It is characterized by a consistent pattern of online or offline gaming behaviours, that results in substantial impairment in various aspects of an individual’s physical and psychological health, social, family, educational, occupational, or other significant areas of functioning [9]. A meta-analysis of IGD prevalence in East Asian regions reported high prevalence in Taiwan (9%), South Korea (11%), Japan (15%) and Mainland China (14%) [10]. Furthermore, IGD is particularly prevalent in adolescents [11], especially among boys [12]. A recent large-sample survey in Hong Kong indicated that 14.1% of secondary students met the criteria for IGD, with 16.4% in boys and 9.5% in girls [13].

Prolonged internet gaming, especially binge gaming, may be a significant risk factor for IGD. The notion of binge refers to a tendency to engage in or repeat a particular behaviour in a short period or single occasion, often accompanied by a loss of control [14]. Binge episodes, including binge drinking, binge eating, and binge gambling, are important behavioural markers, to prevent more severe long-term consequences, such as addiction and severe mental disorders [15,16]. Binge gaming is defined as gaming for five or more hours consecutively [7]. Frequently engaging in binge gaming may implicate that gamers are craving and immersed in the virtual gaming environment, over-rely on the virtual reward systems, have diminished interest in real-life activities and interpersonal interactions, and have sedentary lifestyles. With the outdoor restrictions and lockdown measures during the COVID-19 pandemic, this issue may have risen [17]. There was scarce empirical research examining the prevalence or roles of binge gaming in social, academic, and mental outcomes. We only identified one study on the prevalence or correlates of binge gaming. In a recent study of young Swiss men (*N* = 5,356, Mean age = 28.26), 33.3% of the participants reported engaging in binge gaming at least once within the previous year; 6.1% had binge gaming at least weekly [7]. After adjusting for daily gaming time, binge gaming was significantly negatively associated with life satisfaction and sleep quality, and positively associated with gaming disorder, major depression, and social anxiety disorder [7]. To our knowledge, there were no empirical studies on binge gaming in children and adolescents.

### Study aims

This study aims to examine the prevalence and potential mental, social and educational self-efficacy correlates/consequences of binge gaming among primary and secondary school students in Hong Kong. It is hypothesized that boys would be more likely to have binge gaming than girls. Also, binge gamers would have higher levels of IGD, depression, anxiety, stress, and loneliness, poorer sleep quality, and lower levels of social support and educational self-efficacy, compared to non-binge gamers and non-gamers.

## Materials and methods

### Study design and participants

A cross-sectional school-based survey with convenience sampling was conducted in primary and secondary schools in Hong Kong from June 13, 2022 to December 31, 2022, except for the summer holidays in July and August. In total, 3,154 students and their parents were invited to participate, and 2,770 completed the survey, resulting in a response rate of 87.8%. Chinese students (*N* = 2,770) from five primary schools and four secondary schools in different districts and with different bandings, which are categorized according to academic performance, participated in the study. The inclusion criteria of the current study included (1) being primary or secondary school students; (2) being willing to participate in the research; (3) providing students’ and parents’ informed consent; and (4) being a Chinese speaker.

### Recruitment procedures

With the assistance of the Hong Kong Association for School Discipline and Counselling Teachers, invitation letters with the project introduction were emailed to all government-subsidized primary (*n* = 422) and secondary (*n* = 390) schools in Hong Kong. Teachers at participating schools helped to invite parents and students. The first author explained the significance and logistics of the survey, as well as the principles of voluntariness, anonymity, and confidentiality, to the teachers. The first author provided them with a brief note and instructions and clarified the information over the phone. Students completed the self-administered questionnaire in classroom settings. Written informed consent was obtained from all the participants, including both the students and their parents. Schools distributed parental consent forms to parents for signing either directly or through their internal intranet systems. To ensure data security, only the research team had access to the collected information. A survey delivery note containing clear instructions and step-by-step guidelines for managing the process of distributing and collecting questionnaires was provided to the counselling teachers, who are independent from the research team. No incentive was offered to the participants.

### Measures

#### Binge gaming.

It was assessed by asking the participants whether they have consecutively played five or more hours of internet games in the past month [7]. Answer options included “yes = 1” and “no = 0”.

#### The DSM‑5 IGD symptoms checklist for adolescents (DISCA).

It was used to assess IGD symptoms. It was developed based on the nine DSM-5 IGD criteria [8]. It has been well-validated in Chinese adolescent population, demonstrating high reliability and validity (Cronbach’s alpha is.77) [18]. This brief self-report instrument assesses symptoms of IGD, including preoccupation, tolerance, withdrawal, failed attempts to reduce gaming, deception or lies about gaming, loss of interest in other activities, continued use despite knowledge of adverse consequences, use of gaming as a way to escape or relieve negative emotions, and harm according to DSM-5 criteria. Participants were asked whether they had experienced symptoms in the last 12 months (0 = no, 1 = yes). Consistent with the DSM-5, IGD was defined as meeting at least five of the nine criteria [8]. The Cronbach’s alpha value in the present study was.71.

#### Multidimensional scale of perceived social support (MSPSS).

It was used to measure perceived social support. It consists of 12 items that relate to 3 dimensions: Family, Friends, and Significant others [19]. Each item is scored on a 7-point Likert scale (1 = very strongly disagree to 7 = strongly agree). The total score is the sum of the results for all items, which ranges from 12 to 84, a higher score indicating a higher level of perceived social support. Besides, separate subscales can be used by summing the responses from items in each of the three dimensions. The range of the possible sources for the subscales is between 4 and 28. The Cantonese version of MSPSS demonstrated a high level of internal consistency with a Cronbach’s alpha coefficient of.89 [20]. The Cronbach’s alpha value in the present study was.91.

#### Three-item UCLA loneliness scale.

It was used to measure participants’ loneliness or social isolation [21]. It consists of 3 items, and each item is scored on a 3-point Likert scale (1 = hardly ever; 2 = some of the time; 3 = often). A higher score indicates a higher level of loneliness. The UCLA-3 showed a high degree of reliability and validity in the previous study (Cronbach’s alpha is.87) [22]. The Cronbach’s alpha value in the present study was 0.83.

#### Depression, anxiety and stress scale 21 (DASS-21).

It is a well-established self-report questionnaire to assess individuals’ emotional states of depression, anxiety, and stress [23]. Each of the 3 subscales contains 7 items. The items are rated on Likert scales (0 = did not apply to me at all to 3 = applied to me very much or most of the time). A higher score indicates greater severity or frequency of negative emotional symptoms. To calculate the cut-off, the summed numbers in each subscale need to be multiplied by 2 first. Symptom severity is classified into five categories based on the total scores for each subscale. For depression, scores range as follows: normal (0–9), mild (10–13), moderate (14–20), severe (21–27), and extremely severe (28+). For anxiety, the ranges are normal (0–7), mild (8–9), moderate (10–14), severe (15–19), and extremely severe (20+). For stress, the ranges are normal (0–14), mild (15–18), moderate (19–25), severe (26–33), and extremely severe (34+). In this study, the cut-off scores used were 10 or above for depression, 8 or above for anxiety, and 15 or above for stress, including mild symptoms or higher. Both the English and Chinese versions of the DASS have been used in Hong Kong and have demonstrated strong reliability and validity [24]. The scale demonstrated good internal consistency, with Cronbach’s alpha values of.90,.86, and.88 for the depression, anxiety, and stress subscales, respectively [25]. The Cronbach’s alpha value in the present study was.93.

#### Pittsburgh sleep quality index (PSQI).

It was used to examine participants’ attitudes toward sleep quality over the last 4 weeks [26]. It includes 7 components, including subjective sleep quality, time taken to fall asleep, sleep duration, habitual sleep efficiency, sleep disruptions, use of sleep medication, and daytime dysfunction. The total score ranges from 0 to 21 and each item is scored on a 0–3 Likert scale (0 = no difficulty to 3 = severe difficulty). A higher score indicates a lower quality of sleep. Individuals who scored 5 or greater indicate significant sleep difficulties. The PSQI has a high degree of reliability and validity [27]. The Chinese version of the PSQI demonstrates strong reliability and validity (*r* = .82 –.83) as well as robust test–retest reliability (*r* = .77 –.85) [28]. The Cronbach’s alpha value in the present study was.61.

#### Educational self-efficacy scale (ESES).

It was used to investigate student’s perceptions of their capability to achieve academic results. It consists of five items and all the items were rated by using a 5-point Likert scale (1 = not at all confident to 5 = extremely confident). This scale was used among the Hong Kong Chinese youth population with a good reliability (Cronbach’s alpha value of.87) [29]. A higher score indicates a higher confidence level in achieving academic outcomes [30]. The Cronbach’s alpha value in the present study was.89.

Background information including gender, age, education level, and average time spent on gaming per day during weekdays and weekends, respectively, was reported by the participants.

### Analytical procedure

Participants were divided into three groups, including non-gamers, non-binge gamers, and binge gamers based on whether they played internet games in the past year and had binge gaming. A total of 178 individuals, constituting approximately 6.4% of respondents, were excluded from the analyses due to the absent information of binge gaming. ANCOVA models adjusting for age and gaming time were conducted to identify the relationships between the three groups and mental, social, and academic outcomes. The analyses were stratified by gender. Statistical significance was set at the.05 level. SPSS 27.0 Statistics for Windows was used for all statistical analyses.

### Ethical considerations

Written informed consent was obtained from all participants, including the students, with parental consent secured from their parents. The study did not contain clinical studies or patient data. The study procedures were carried out in accordance with the Declaration of Helsinki. Ethical approval was obtained from the Survey and Behavioural Ethics Committee of the corresponding author’s university (SBRE-21–0731).

## Results

The final analyses included 2,592 students. The sample included 1,404 boys with a mean age (+/-SD) of 11.99 + /- 2.415 years and 1,188 girls with a mean age (+/-SD) of 11.77 + /- 2.473 years in Table 1. The overall prevalence of binge gaming in the study sample is approximately 31.7%. Boys are more likely to have binge gaming (boys 38.3%, girls 24.0%, *p* < .001), but less likely to have poor sleep quality (boys 58.6%, girls 65.5%, *p* < .001) than girls. No gender differences were found in the rate of depression (boys 32.9%, girls 33.8%, *p* > .05), anxiety (boys 40.0%, girls 43.2%, *p* > .05), stress (boys 23.8%, girls 26.6%, *p* > .05).

**Table 1:** Descriptive statistics by gender (*N* = 2,592).

	Boys		Girls	
Variables	*N*	Mean (*SD*)/%	*n*	Mean (SD)/%
Gender	1,404	54.2%	1,188	45.8%
Age		11.99(2.415)		11.77(2.473)
Level of education				
Primary school	656	46.7%	630	53.0%
Secondary school	748	53.3%	558	47.0%
Time spent gaming (TSG)				
Weekdays				
No gaming	425	30.3%	569	47.9%
1 hour	389	27.7%	343	28.9%
2 hours	288	20.5%	142	12.0%
3 hours	135	9.6%	73	6.1%
4 hours	74	5.3%	25	2.1%
5 hours	40	2.8%	15	1.3%
6 hours	18	1.3%	5	0.4%
More than 6 hours	35	2.5%	16	1.3%
Weekends				
No gaming	203	14.5%	346	29.1%
1 hour	261	18.6%	276	23.2%
2 hours	285	20.3%	267	22.5%
3 hours	215	15.3%	120	10.1%
4 hours	138	9.8%	68	5.7%
5 hours	96	6.8%	45	3.8%
6 hours	68	4.8%	18	1.5%
More than 6 hours	138	9.8%	48	4.0%
Gaming behaviors				
Non-gamers	87	6.2%	164	13.8%
Binge gamers	538	38.3%	285	24.0%
Non-binge gamers	779	55.5%	739	62.2%
Internet gaming disorder (IGD)	226	16.1%	100	8.4%
Depression	462	32.9%	404	33.8%
Anxiety	561	40.0%	513	43.2%
Stress	334	23.8%	316	26.6%
Poor sleep quality^a^	823	58.6%	778	65.5%
Loneliness	/	4.73(1.71)	/	5.03(1.77)
Educational self-efficacy	/	15.98(4.93)	/	15.42(4.75)

Cut-off for DASS: mild or above levels. Non-gamers refer to students who did not engage in gaming in the last year. Binge gamers refer to students who played games for 5 or more consecutive hours at least once in the past month. Non-binge gamers refer to students who played games in the past month but did not meet the criteria for binge gaming.

^a^Missing data

https://doi.org/10.5281/zenodo.15356460

For boys, after adjusting for age and gaming time, binge gaming was significantly associated with levels of IGD, depression, anxiety, stress, sleep quality, and educational self-efficacy. Compared to non-binge gamers, binge gamers were more likely to have IGD (mean difference (MD) =.248, 95% *CI* = [.135,.361]), depression (*MD* = .191, 95% *CI* = [.072,.310]), anxiety (*MD* = .206, 95% *CI* = [.089,.322]), stress (*MD* = .155, 95% *CI* = [.035,.276]) and less likely to have good sleep quality (*MD* = .209, 95% *CI* = [.086,.332]) and educational self-efficacy (*MD* = −.114, 95% *CI* = [−.233,.006]). Compared to non-binge gamers, non-gamers had less stress (*MD* = −.259, 95% *CI* = [−.479, −.038]), less loneliness (*MD* = −.245, 95% *CI* = [−.462, −.028]), and more educational self-efficacy (*MD* = .299, 95% *CI* [.081,.517]). Similar results were found in ANCOVA adjusting for only age in Table 2.

**Table 2:** Associations of binge gaming (BG) and social, academic, mental health variables.

	Boys		Girls	
	No gaming	Binge gaming	No gaming	Binge gaming
IGD symptoms				
Adjusted for age	na	**.522** **[.415,.628]** ^***^	Na	**.609** **[.498,.721]** ^***^
Adjusted for age and TSG	na	**.248** **[.135,.361]** ^***^	Na	**.312** **[.196,.428]** ^***^
Depression symptoms				
Adjusted for age	−.159 [-.375,.056]	**.218** **[.110,.325]** ^***^	**−.283** **[-.448, -.118]** ^***^	**.473** **[.340,.606]** ^***^
Adjusted for age and TSG	−.147 [-.364,.070]	**.191** **[.072,.310]** ^**^	**−.234** **[-.401, -.068]** ^**^	**.363** **[.217,.508]** ^***^
Anxiety symptoms				
Adjusted for age	−.185 [-.397,.026]	**.242** **[.136,.347]** ^***^	**−.248** **[-.415, -.080]** ^**^	**.464** **[.329,.598]** ^***^
Adjusted for age and TSG	−.169 [-.381,.044]	**.206** **[.089,.322]** ^***^	**−.202** **[-.371, -.033]** ^*^	**.359** **[.211,.507]** ^***^
Stress symptoms				
Adjusted for age	**−.274** **[-.492, -.055]** ^*^	**.187** **[.078,.297]** ^***^	**−.236** **[-.400, -.072]** ^**^	**.401** **[.270,.533]** ^***^
Adjusted for age and TSG	**−.259** **[-.479, -.038]** ^*^	**.155** **[.035,.276]** ^*^	**−.202** **[-.367, -.037]** ^*^	**.324** **[.179,.468]** ^***^
Poor sleep quality levels				
Adjusted for age	**−.257** **[-.482, -.033]** ^*^	**.274** **[.163,.385]** ^***^	−.047 [-.220,.125]	**.399** **[.260,.538]** ^***^
Adjusted for age and TSG	−.222 [-.448,.004]	**.209** **[.086,.332]** ^***^	.022 [-.151,.195]	**.233** **[.081,.386]** ^**^
Social support levels				
Adjusted for age	.126 [-.100,.353]	−.026 [-.139,.087]	.090 [-.068,.248]	**−.266** **[-.393, -.139]** ^***^
Adjusted for age and TSG	.101 [-.127,.328]	.029 [-.096,.154]	.056 [-.104,.215]	**−.188** **[-.327, -.048]** ^**^
Loneliness levels				
Adjusted for age	**−.263** **[-.479, -.048]** ^*^	.064 [-.044,.171]	**−.250** **[-.419, -.082]** ^**^	**.295** **[.159,.430]** ^***^
Adjusted for age and TSG	**−.245** **[-.462, -.028]** ^*^	.024 [-.095,.143]	**−.216** **[-.387, -.046]** ^*^	**.217** **[.068,.366]** ^**^
Educational self-efficacy levels				
Adjusted for age	**.346** **[.128,.563]** ^**^	**−.214** **[-.322, -.105]** ^***^	**.247** **[.091,.402]** ^**^	**−.295** **[-.421, -.170]** ^***^
Adjusted for age and TSG	**.299** **[.081,.517]** ^**^	**−.114** **[-.233,.006]** ^*^	**.185** **[.029,.341]** ^*^	**−.154[−.291, −.018]** ^*^

The results of ANCOVA (MD [95% CI]). Reference group: gaming without binge. The criterion variables have been z-standardized, and the coefficients represent mean differences in standard deviations between gender and the mental health indicators. TSG: time spent on gaming. For non-gamers, as the IGD score is consistently 0, no coefficient was estimated (denoted as ‘na’). Coefficients reaching different levels of significance are denoted in bold: ^*^ *p* < .05, ^**^ *p* < .01, ^***^ *p* < .001.

https://doi.org/10.5281/zenodo.15356460

Among girls, after adjusting for age and gaming time, binge gaming was significantly associated with levels of IGD, depression, anxiety, stress, sleep quality, loneliness, educational self-efficacy, and social support. Compared to non-binge gamers, binge gamers showed significantly higher levels of IGD (*MD* = .312, 95% *CI* = [.196,.428]), depression (*MD* = .363, 95% *CI* = [.217,.508]), anxiety (*MD* = .359, 95% *CI* = [.211,.507]), stress (*MD* = .324, 95% *CI* = [.179,.468]), loneliness (*MD* = .217, 95% *CI* = [.068,.366]), and lower levels of educational self-efficacy (*MD* = −0.154, 95% *CI* = [−.291, −.018]), sleep quality (*MD* = .233, 95% *CI* = [.081,.386]), and social support (*MD* = −.188, 95% *CI* = [−.327, −.048]). Compared to non-binge gamers, non-gamers had lower levels of depression (*MD* = −.234, 95% *CI* = [−.401, −.068]), anxiety (*MD* = −.202, 95% *CI* = [−.371, −.033]), stress (*MD* = −.202, 95% *CI* = [−.367, −.037]), loneliness (*MD* = −.216, 95% *CI* = [−.387, −.046]), and more educational self-efficacy (*MD* = .185, 95% *CI* = [.029,.341]) in Table 2. Similar results were found in ANCOVA adjusting for only age.

## Discussion

To the best of our knowledge, this study represents the first investigation of gender differences in the prevalence and correlates of binge gaming among children and adolescent populations. The data supported most hypotheses, while intriguing findings were also identified. Overall, both boys and girls who engaged in binge gaming showed significantly higher levels of IGD, depression, anxiety, and stress than non-binge gamers. They also reported poorer sleep quality and lower educational self-efficacy. However, non-gamers demonstrated lower levels of stress and loneliness, and higher levels of educational self-efficacy than non-binge gamers. The result is consistent with the previous study [7] and highlights the need to limit binge gaming behaviors to prevent negative mental consequences, such as depression and anxiety symptoms. Binge gaming might signify an initial phase in the progression of IGD and other severe social, physical and mental health issues. With reference to the dynamic model of disease progression which was originally developed to illustrate the progression of prostate cancer from the early sign, the point of diagnosis, through the final phase of life, assessing the predictive signs and symptoms of disease progression can guide clinical decision-making at each state of disease progression for disease prevention and contribute personalized, data-driven prevention and treatment strategies to improve patient wellbeing [31]. Our finding provides preliminary evidence to the application of the model in gaming behaviours. This model outlines the evolving states from non-gamer to recreational gamer (gaming primarily for positive reinforcement, i.e., enjoyment), habitual gamer (gaming regularly for less than five hours consecutively, generally associated with a lower risks of negative consequences), binge gamer (gaming for five or more hours consecutively, often associated with higher risks of negative consequences), and potentially leading to a disordered gamer (gaming that meets the criteria for diagnosable IGD, with significant impacts on one’s life). The crux of this model posits that binge gaming may precede the development of IGD, and can be applied to facilitate the early detection and prevention of IGD and related mental disorders. However, it is also important to acknowledge that other potential risk factors, such as Attention-deficit/hyperactivity disorder [32], Autistic Spectrum Disorders [33], and parental factors [34], may contribute to IGD. Therefore, further investigation to validate this state model while considering these additional risk factors and exploring the pattern of binge gaming more comprehensively (e.g., frequency in different time frames, in which contexts, daytime versus nighttime), which will provide a better understanding of binge gaming and its roles in mental health and well-being.

Significant gender differences were also identified. First, we found that boys (38.3%) had higher rates of binge gaming than girls (24.0%). Similar gender differences were also found in other binge behaviors, such as binge drinking (males: 32.6% versus females: 12.8%) [35]. These behaviours are male-dominated addictive behaviours that are also initiated and maintained due to male peer influence and gender norms [36,37]. In the context of gaming, boys ages 14 and older are up to five times more likely than girls to be involved in gaming, which is influenced by several factors. Boys have the tendency of spending more time on gaming and often use it as a tool for socialization to further foster the bonding with peers, reinforcing their engagement. In addition, boys find more appealing to competitive and complex gameplay, which fits into the masculine gaming culture that encourages aggression and competition. This sort of culture is also a barrier to girls engaging in such video gaming [36]. However, no evidence supports that risks for IGD in males are significantly higher than in females. Further studies should investigate gender differences beyond attitudes towards internet gaming, but also identify structure and differentiated responses in the brain circuits governing attention, decision-making, and sensory-motor coordination [38].

Secondly, among girls, but not boys, binge gamers experienced significantly greater loneliness and lower social support, than non-binge gamers. This suggests a potential gender-specific social impact of binge gaming. It is plausible that gaming is not dominated by females, and thus female binge gamers could not develop meaningful interpersonal relationships through prolonged gaming [39]. The motives and triggers of binge gaming may be also different between girls and boys. Girls may engage in binge gaming because they are lonely and lack social support in real life, while boys are likely to binge game because their peers have the same gaming pattern and play together [37]. It highlights the need for extra attention to girls’ social needs to facilitate social support and cultivate a sense of companionship, which may help mitigate the risk of developing binge gaming and prevent its adverse consequences.

It is interesting to find that even non-binge gamers showed significantly poorer emotional health with higher levels of stressful, depressive, and anxiety symptoms, compared to non-gamers in girls but not boys. During online multiplayer games, female gamers’ distress and anxiety is often attributed to encounter not only general harassment, but also sexual harassment [40]. A longitudinal study on adolescent mental health reported that female adolescents aged from 12 to 15 years showed much higher depressive and anxiety symptoms compared to male adolescents [41]. It seems that girls generally showed relatively greater challenges in emotional health than boys. Therefore, it is crucial to train girls’ emotional regulation skills to improve emotional well-being, which may help to prevent binge gaming.

### Practical implications

The identified significant associations between binge gaming and various mental, social, and academic consequences after adjusting for average gaming hours per day highlight the necessity to understand better the gaming patterns instead of detecting only gaming hours. Binging or not may add additional value in addition to gaming hours to better predict mental and gaming disorders and help to identify the high-risk groups for early prevention. While prevention work could focus on educational programs to raise awareness of the risks of binge gaming and promote healthy gaming habits, interventions such as Motivational Interviewing (MI) and Cognitive Behavioral Therapy (CBT) could be used for individuals with problematic gaming behaviors. MI, a psycholinguistic counselling approach, emphasizes evoking an individual’s intrinsic motivation and fostering personal growth by exploring personal values and reasons for change [42]. It could serve as a prelude intervention with other intensive therapies and stand-alone treatment to motivate gamers to have regulated breaks during gaming. CBT, which aims to enhance skills of time management, self-regulation, emotional management, and problem-solving may help to manage craving and binge gaming [43]. The combination of MI and CBT displays substantial potential as a comprehensive intervention for addressing binge gaming [44]. Besides, mindfulness and acceptance commitment therapy [45] which are third-wave therapies and trans-diagnostically address emotional distress may be also promising to reduce binge gaming. Future interventions should also be gender-specific as our results showed significant gender differences in binge gaming and its correlates. Female gamers regardless of binge or not may suffer more negative experiences than male gamers and have long been an under-examined population. Tailored psychosocial interventions and prevention programs should address the social and interpersonal needs of female gamers more effectively.

### Limitations

Several limitations should be addressed in future studies. The cross-sectional design could not demonstrate the causal relationships between variables. The use of a convenience sample in Hong Kong limited the generalizability of the findings due to variations in cultures, school demographics, policies, resources, and academic levels. We measured binge gaming using a single-item and self-reported question. A better developed and validated scale to better capture the gaming pattern and frequency and objective data (e.g., digital device record) is needed. Longitudinal studies with random sampling, cross-cultural data, and well-validated scales are recommended to better understand binge gaming and its causes and consequences.

## Conclusion

This study represents the first piece of evidence on binge gaming in adolescent and student populations, shedding light on an area that has received limited scholarly attention. The findings reveal that boys had higher rates of binge gaming than girls. Particularly, female binge gamers experienced greater loneliness and lower social support, which showed a gender-specific social impact. While binge gaming is in the initial stage of the investigation, these findings lay the groundwork for future empirical research to explore its potential as a diagnostic criterion, its roles, mechanisms contributing to IGD, as well as its academic, social, and health consequences. The observed patterns and frequency of binge gaming underscore its significance in relation to IGD and associated mental health issues. Gender-specific experiences related to binge gaming should be further explored. A well-validated screening tool is warranted to effectively identify related problems and provide appropriate treatment for binge gaming. Harm reduction strategies aim to minimize the harmful effects of binge gaming behaviors, such as encouraging regular breaks and limiting prolonged gaming sessions, may help prevent binge gaming and mitigate the negative consequences.

## Supporting information

S1 Data (CSV)
